# Pancreatic Stellate Cells and Metabolic Alteration: Physiology and Pathophysiology

**DOI:** 10.3389/fphys.2022.865105

**Published:** 2022-03-15

**Authors:** Shin Hamada, Ryotaro Matsumoto, Atsushi Masamune

**Affiliations:** Division of Gastroenterology, Tohoku University Graduate School of Medicine, Sendai, Japan

**Keywords:** pancreatic stellate cells, pancreatic cancer, fibrosis, PSC activation, metabolism

## Abstract

Pancreatic stellate cells play a pivotal role in the development of pancreatic fibrosis. A wide variety of external stimuli can cause PSC activation accompanied by metabolic changes, which alters the tissue microenvironment by producing extracellular matrix proteins, cytokines, growth factors, and other mediators. Several metabolites aggravate fibrosis and inflammation by acting as key activating factors for PSCs. In other words, PSCs sense systemic metabolic changes. The detrimental effects of PSC activation on normal pancreatic cells, especially islet cells, further complicate metabolic imbalance through the dysregulation of glucose metabolism. PSC activation promotes cancer by altering the metabolism in pancreatic cancer cells, which collaborate with PSCs to efficiently adapt to environmental changes, promoting their growth and survival. This collaboration also contributes to the acquisition of chemoresistance. PSCs sequester chemotherapeutic agents and produce competing molecules as additional resistance mechanisms. The application of these metabolic targets for novel therapeutic strategies is currently being explored. This mini-review summarizes the role of PSCs in metabolic regulation of normal and cancerous cells.

## Introduction

Pancreatic stellate cells (PSCs) play pivotal roles in the development of fibrosis through the production of extracellular matrix (ECM) proteins, growth factors, and cytokines (Erkan et al., 2012). PSCs contribute to the pathogenesis of pancreatitis and pancreatic cancer by modifying the cellular microenvironment (Masamune and Shimosegawa, 2015), thereby contributing to the development of pancreatic fibrosis, which creates hypoxia, poor blood supply, and mechanical barriers. These structural and environmental changes have a substantial impact on the cellular functions of normal pancreatic cells, leading to tissue damage, and exocrine and endocrine insufficiency. However, in pancreatic cancer cells, activation of PSCs leads to the establishment of a pro-cancer microenvironment that contributes to the intractable nature of pancreatic cancer (Masamune and Shimosegawa, 2015). Furthermore, recent studies have identified metabolic alterations in PSCs, normal pancreatic cells, and pancreatic cancer cells that affect cellular functions. Here, we review several key metabolic changes related to PSCs in the pathogenesis of pancreatitis and pancreatic cancer. In addition, we discuss the possibilities for therapeutic applications based on cancer-promoting PSC activation.

## PSC Activation

Within the normal pancreas, quiescent PSCs with vitamin A-containing lipid droplets reside around acinar cells (Haeberle et al., 2018). Pancreatic injury and inflammation activate PSCs, which are then characterized by myofibroblast-like phenotypes such as α-smooth muscle actin (α-SMA) expression, ECM production, and increased proliferation. A wide variety of growth factors, cytokines, and environmental stressors are involved in PSC activation. For example, platelet-derived growth factor and transforming growth factor β (TGFβ) treatment can activate downstream signaling pathways, leading to PSC activation (Duner et al., 2010). Interaction between pancreatic cancer cells and PSCs also activates PSCs (Masamune and Shimosegawa, 2015).

Induction of acute pancreatitis or oncogenic mutations, such as *K-ras*, leads to an increase in the reactive oxygen species (ROS; Wang et al., 2015; He et al., 2021), and increased oxidative stress activates PSCs. The administration of hydrogen peroxide increases α-SMA expression, migration, and invasive capacity of PSCs (Yan et al., 2018). A PSC-activating cytokine such as interleulin-1β and a vasoactive peptide angiotensin II increase intracellular ROS in PSCs, which are essential for activation (Masamune et al., 2008b). The formation of dense fibrotic plaques causes tissue hypoxia, which also leads to PSC activation, resulting in increased migration and production of collagen I, vascular endothelial growth factor, and connective tissue growth factor (Masamune et al., 2008a; Eguchi et al., 2013). Expression of connective tissue growth factor in pancreatic cancer tissue is also increased (Eguchi et al., 2013). Indeed, pancreatic cancer cells have a characteristic gene expression signature related to hypoxia (Chen et al., 2021).

**Figure 1:**
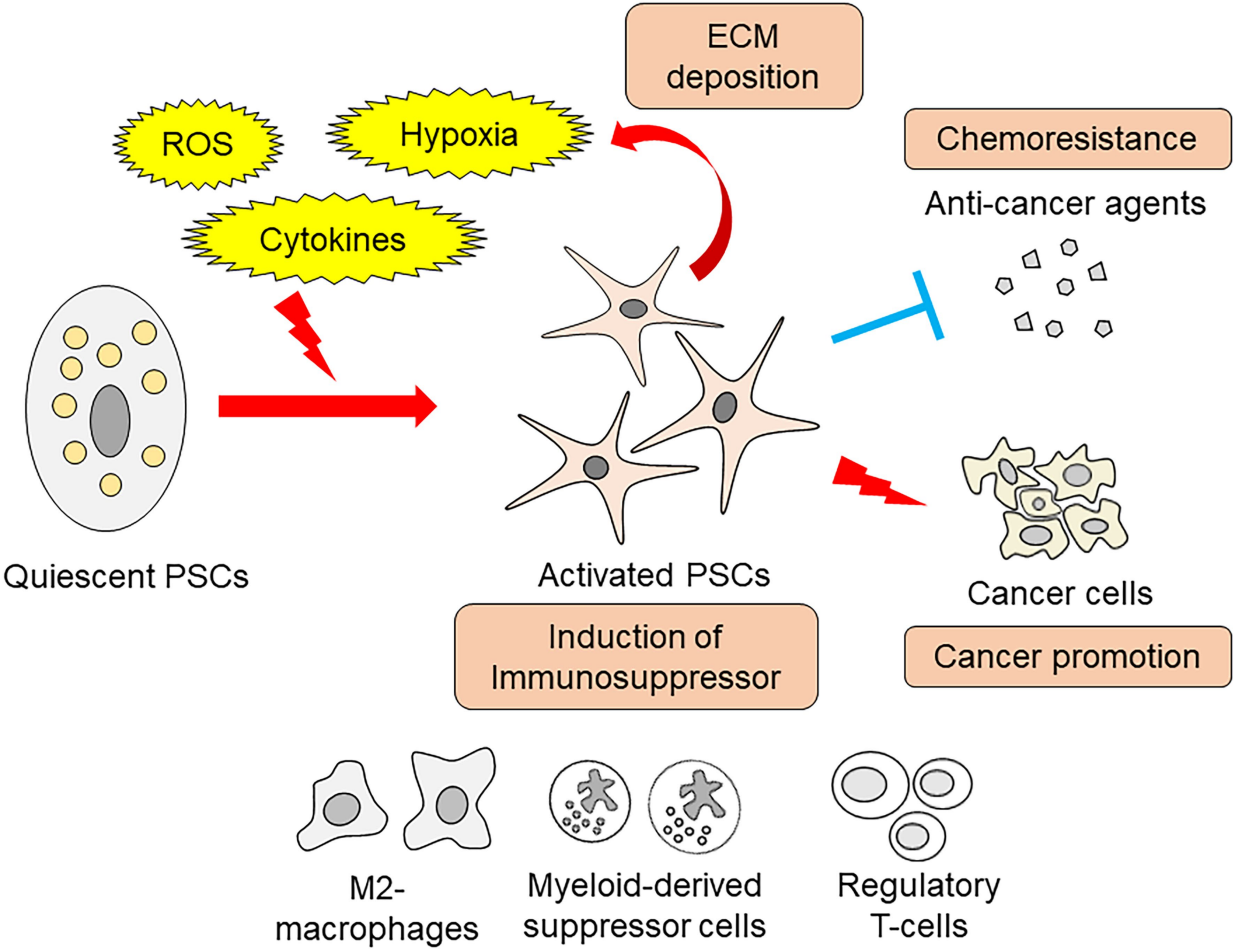
Schema of PSCs roles in pancreatitis and pancreatic cancer.

Activated PSCs promote immunosuppression and contribute to pancreatic cancer progression. Orthotopic implantation of pancreatic cancer cells with PSCs increased the number of regulatory T-cells, M2-type macrophages, and myeloid-derived suppressor cells within implanted tumors (Li et al., 2020). Another study identified that activated PSCs reduce cytotoxic T-cell infiltration into pancreatic cancer tissue, hampering their effect on cancer cells (Garg et al., 2018). The cancer-promoting roles of PSCs have been extensively studied, and a wide variety of effects have been recognized. PSC-derived hepatocyte growth factor induces nerve growth factor expression in pancreatic cancer cells, thereby increasing the invasive capacity and affinity to the nerve (Nan et al., 2019). This paracrine hepatocyte growth factor also activates the c-Met/Akt pathway in pancreatic cancer cells, resulting in resistance to the chemotherapeutic agent gemcitabine (Xu et al., 2020). Recent studies have uncovered metabolic and nutritional changes underlying these pancreatic cancer-promoting cell-to-cell interactions. Schematic view of PSCs’ roles in pancreatitis and pancreatic cancer is summarized in Figure 1.

## Metabolic Alterations in PSCs

The activation of PSCs involves various changes in the metabolic processes. On the other hand, several metabolites activate PSCs, leading to fibrogenesis. Alcohol is a major cause of acute and chronic pancreatitis (Masamune et al., 2020a,b). Alcohol metabolism involves two types of metabolic pathways in the pancreas: oxidative and non-oxidative (Srinivasan et al., 2021). Oxidative alcohol metabolism increases ROS production, while non-oxidative metabolism produces fatty acid ethyl esters (FAEEs), especially in pancreatic acinar cells. Both metabolites injure acinar cells, leading to pancreatitis. Ethanol and acetaldehyde increased collagen I production in PSCs. Induction of the inflammatory cytokine, interleukin-8, mainly relied on ethanol. FAEEs, such as palmitic acid ethyl ester, increased activator protein-1-dependent transcriptional activity. Palmitic acid ethyl ester also activates mitogen-activated protein kinases (MAPKs), such as c-Jun N-terminal kinase, p38 MAPK and extracellular signal-regulated kinase (Masamune et al., 2010). In addition to alcohol, a high-fat diet also alters the cellular function of PSCs. PSCs derived from high-fat diet-fed rats showed elevated expression of the transient receptor potential family member, transient receptor potential vanilloid type 4 (TRPV4). A continuous high-fat diet increased α-SMA- and TRPV4-positive PSCs within the pancreas, which was accompanied by increased fibrosis. PSCs isolated from high-fat diet-fed rats showed an increased response to arachidonic acid-induced calcium mobilization in a TRPV4-dependent manner (Zhang et al., 2013). These studies confirmed that metabolic and nutritional changes cause PSC activation.

Diabetes mellitus (DM)-related metabolites are also involved in PSC activation. Advanced glycation end products (AGEs) are formed by non-enzymatic glycation and oxidation of proteins or lipids. Increased AGE formation is observed in various pathogenic conditions with elevated glucose levels, such as DM (Salazar et al., 2021). AGEs are recognized by the cell-surface receptor, receptor for advanced glycation end products (RAGE), which alters cellular functions. A high-fat diet induced an increase in *TGFβ*, *α-SMA*, and *collagen 1A1* mRNA in wild-type mice, but not in PSCs derived from *RAGE-null* mice. Conditioned medium of AGE-stimulated PSCs induced epithelial-to-mesenchymal transition (EMT) in human pancreatic cancer cell lines, suggesting that RAGE signaling contributes to the cancer-promoting role of activated PSCs. The ratio of RAGE-positive cells correlated with the degree of α-SMA expression around pancreatic intraepithelial neoplasm, a precancerous lesion, found in surgically resected human pancreatic cancer specimens (Uchida et al., 2021).

Activation of PSCs alters the cellular metabolism of PSCs. PSCs cultured in 10% fetal bovine serum-supplemented medium showed increased expression of *α-SMA* and *collagen 1A1* mRNA. These activated PSCs also revealed an increase in glutaminase 1 (GLS1), which is a rate-limiting enzyme for glutaminolysis. Increased glutaminolysis provides more α-ketoglutarate to the tricarboxylic acid cycle, resulting in increased energy production and synthesis of nucleic acids, amino acids, and lipids (Zhang et al., 2022). Induction of GLS1 in PSCs is dependent on the Yap-Myc signal, which has been shown to govern metabolic homeostasis in pancreatic cancer cells (Murakami et al., 2019). Overexpression of YAP in primary cultured PSCs significantly increased GLS1 expression, which was attenuated by Myc inhibitor treatment (Zhang et al., 2022).

**Figure 2:**
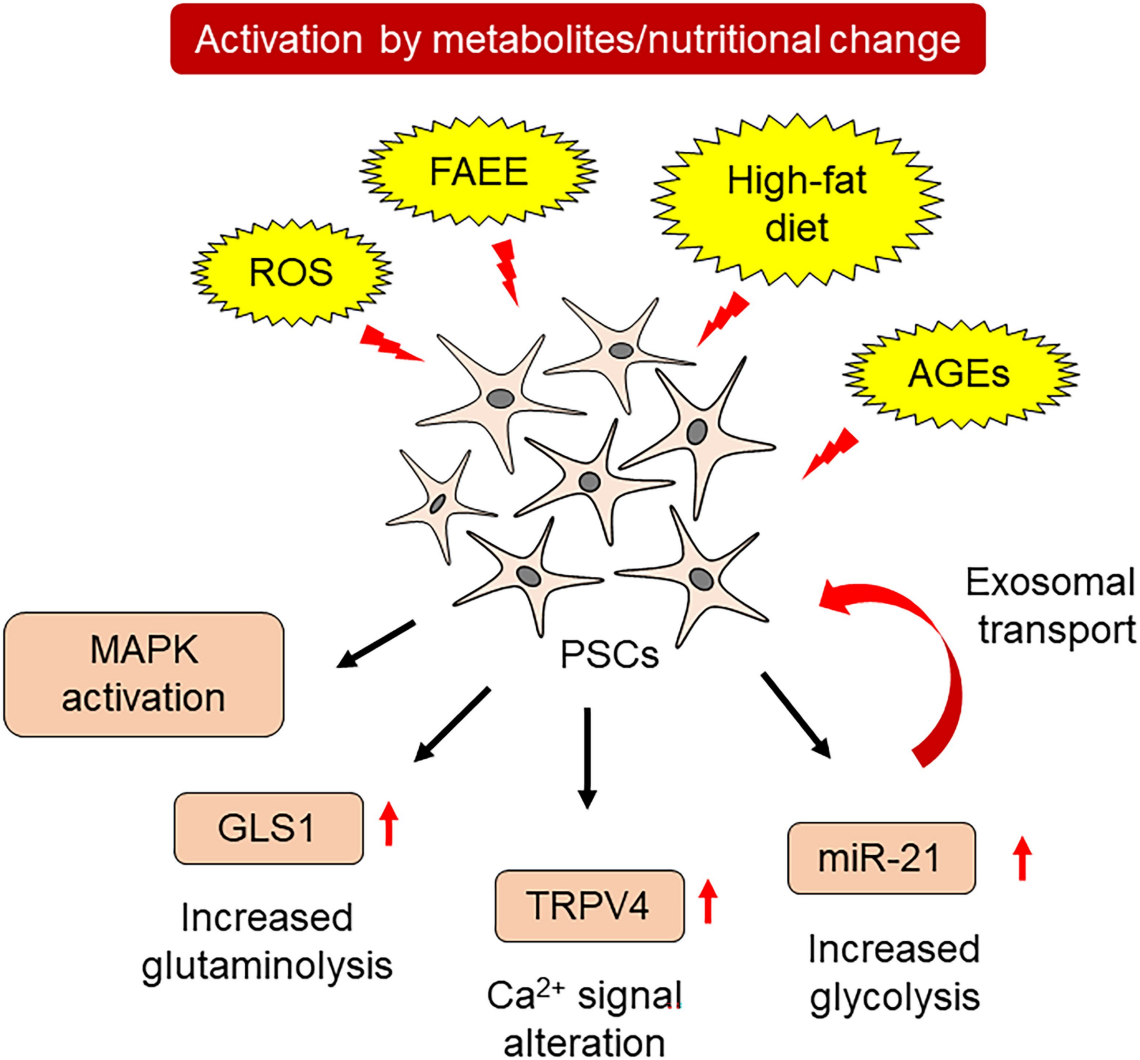
Metabolic changes and PSC activation.

Another class of molecules contributes to the metabolic reprogramming of PSCs. MicroRNAs consist of 21–24 nucleotides. These single-stranded non-coding RNAs regulate mRNA translation and affect cellular functions (Li et al., 2021a). The activation of PSCs by hydrogen peroxide was reversed by resveratrol, an antioxidant. Hydrogen peroxide increased the expression of miR-21 in PSCs. Inhibition of miR-21 also attenuated hydrogen peroxide-induced cellular migration of PSCs. Together with these changes, resveratrol and miR-21 inhibition decreased lactate production, a hallmark of glycolytic activity, accompanied by decreased expressions of glucose transporter 1, hexokinase 2, pyruvate kinase M2, and lactate dehydrogenase A (Yan et al., 2018). miR-21 is also released from PSCs by exosomes, an extracellular vesicle of approximately 100 nm in diameter that is protected by a lipid bilayer (Charrier et al., 2014). These miR-21-containing exosomes are exported to other PSCs, which mediate metabolic reprogramming and act as feedforward loops for PSC activation. Metabolic changes in PSCs involve various downstream effects summarized in Figure 2.

## Effects of Metabolic Changes in PSCs and Normal Pancreatic Cell Function

Altered metabolic status of PSCs affects normal pancreatic cell function. For example, hypoxia-induced PSC activation causes islet β-cell death. The antioxidant N-acetylcysteine (NAC) attenuates this effect, suggesting that hypoxic PSCs increase oxidative stress (Kim et al., 2020). The presence of activated PSCs also inhibits differentiation of fetal islet–epithelial cell clusters into insulin-positive cells, indicating suppressive effects on islet cells (Li et al., 2021b). High glucose conditions, derived from DM, activate PSCs by elevating oxidative stress (Ryu et al., 2013). Transplantation of activated PSCs into the pancreas reduced β-cell mass in the DM model, Goto-Kakizaki rats. This treatment increased islet fibrosis, and conditioned medium derived from PSCs with high glucose conditions significantly decreased the viability of the islet cell line INS-1, indicating that activated PSCs and high glucose additively compromised islet function (Zha et al., 2014). Similar to DM, a high-fat diet also affects islet function *via* PSC activation. In rats fed a high-fat diet showed increased amount of free fatty acids in the islets, accompanied by more intense islet fibrosis. Treatment of PSCs with palmitic acid increased the activation markers of PSCs, α-SMA, and collagen I. Overexpression of sterol regulatory element-binding protein-1c in PSCs suppressed PSC activation by palmitic acid, as well as detrimental effects on islet cells. This study highlights the importance of adipogenic regulation in PSCs for the maintenance of quiescence (Zhou et al., 2019). Vitamin A-containing lipid droplets are a feature of quiescent PSCs (Haeberle et al., 2018). Administration of a vitamin A-deficient diet to mice for 12 weeks significantly reduced pancreatic vitamin A levels, along with a reduction in C-peptide expression by immunofluorescence. Vitamin A-deficient mice showed impaired glucose tolerance and reduced islet size. Retinol treatment of isolated PSCs from islets reduced PSC activation (Zhou et al., 2020). In addition to islet cells, acinar cell viability is affected by the exacerbation of fibrosis. Induction of chronic pancreatitis by repeated caerulein injection, a cholecystokinin analog, enhanced fibrosis in mice with streptozotocin-induced DM. Increased infiltration of inflammatory cells, deposition of collagen I, and atrophy of the pancreatic parenchyma were observed, with enhanced acinar cell death (Zechner et al., 2014). These lines of evidence suggest that altered metabolic status could form a feedforward loop with inflammatory changes, leading to impaired normal cellular functions.

## Metabolic and Functional Alterations of PSCs in Pancreatitis and Pancreatic Cancer

The cancer-promoting roles of PSCs have attracted great attention, and many studies have been carried out with respect to metabolic changes. PSCs act as a source of nutrition for pancreatic cancer cells under a severe microenvironment. A previous study identified that metabolites from PSCs increase the oxygen consumption ratio of pancreatic cancer cells. PSC-derived alanine was found to be responsible for enhanced biosynthesis, resulting in growth advantages. Alanine production from PSCs depends on autophagy, which is evident in PSCs under normal culture conditions (Sousa et al., 2016). The following study identified the orchestrated transport of alanine in PSCs and pancreatic cancer cells, which was mediated by specific transporters. For alanine excretion, PSCs utilize solute carrier family (SLC) 1A4. Alanine uptake in pancreatic cancer cells is dependent on SLC38A2. In particular, inhibition of SLC38A2 caused a metabolic crisis in pancreatic cancer cells and suppressed proliferation, suggesting the possibility of therapeutic intervention (Parker et al., 2020). PSCs also alter amino acid metabolism in pancreatic cancer cells. Serum samples from patients with pancreatic cancer contained higher concentrations of branched chain amino acids (BCAAs). Enzymes involved in BCAA metabolism were highly expressed in pancreatic cancer cells, which were further altered by co-culture with PSCs. Inhibition of branched-chain ketoacid dehydrogenase kinase attenuated subcutaneous tumor growth in immunodeficient mice, indicating a pivotal role of BCAA metabolism during cancer progression (Jiang et al., 2021). Pancreatic cancer cells and PSCs express interleukin-17B receptor (IL-17RB), and co-culture of pancreatic cancer cells and PSCs increased IL-17RB in PSCs. Interleukin-17B-containing exosomes were released from pancreatic cancer cells, which increased IL-17RB in PSCs. An increase in IL-17RB inhibited mitophagy in PSCs, leading to increased oxidative phosphorylation in PSCs. PSCs overexpressing IL-17RB significantly promoted tumor formation of MiaPaCa-2 pancreatic cancer cells in immunodeficient mice by co-transplantation. PSCs with IL-17RB overexpression increased mitochondrial respiration and decreased glycolysis in pancreatic cancer cells, possibly acting as a modifier of cancer cell metabolism (Li et al., 2021c).

**Figure 3:**
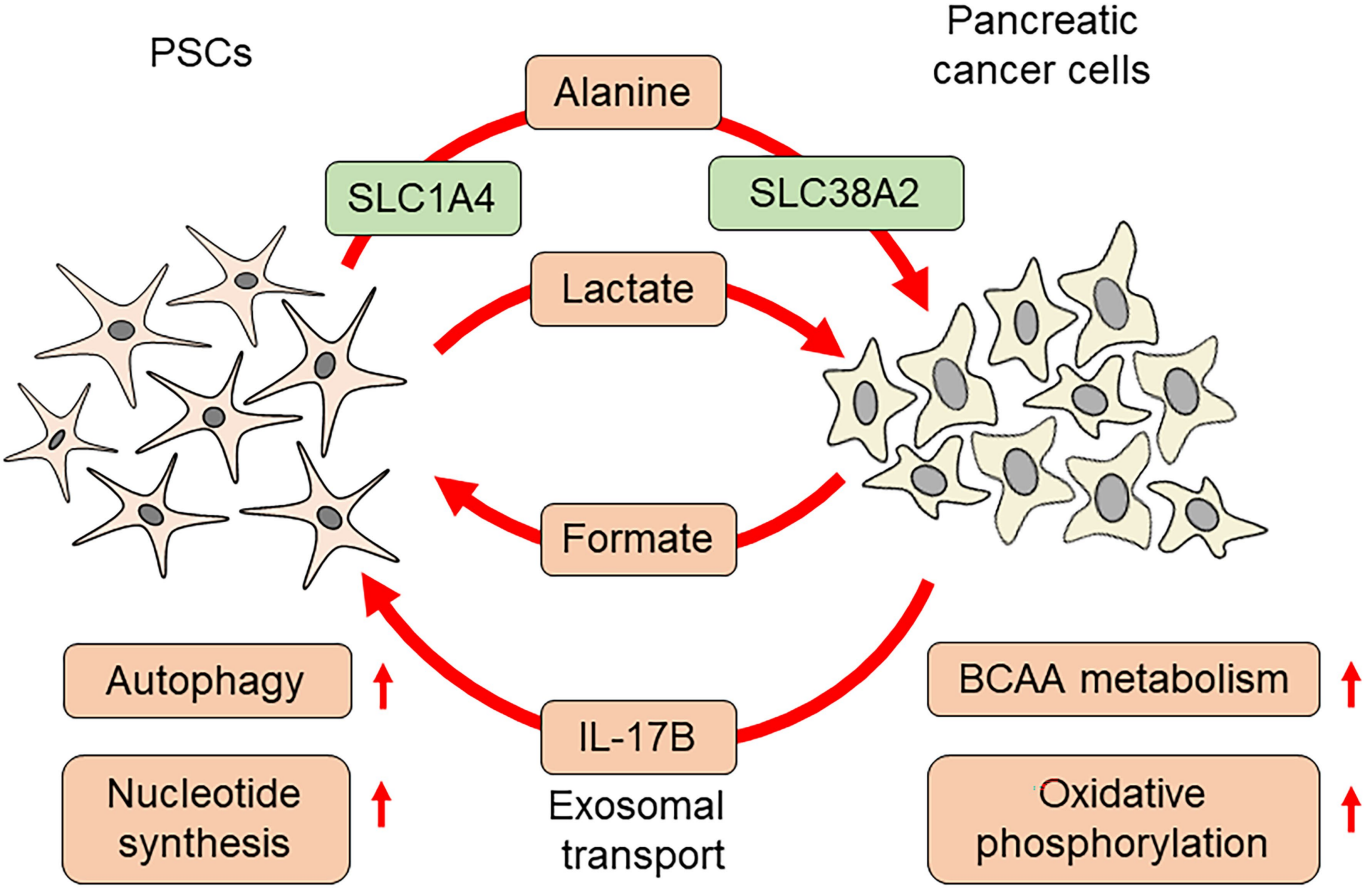
Pancreatic cancer cell metabolism affected by PSCs.

Metabolic coupling between pancreatic cancer cells and PSCs is also important for coordinated cell-to-cell interactions. Reduced expression of Cav-1, a structural component of caveolae, in pancreatic cancer stroma is associated with poor survival. Cav-1 repression promoted ROS production, which further decreased Cav-1. This process was accompanied by increased glycolysis, leading to the accumulation of lactate. These changes, coupled with increased uptake of lactate by pancreatic cancer cells *via* monocarboxylate transporter 1, enabled increased oxidative phosphorylation. Disruption of this coupling by NAC administration efficiently inhibited tumor growth, providing a novel therapeutic target (Shao et al., 2020). Metabolic changes in pancreatic cancer cells can also be coupled with metabolic changes in PSCs. Indoleamine 2,3-dioxygenase-1 (IDO1) is an enzyme that catalyzes the conversion of tryptophan to formyl-kynurenine. Higher expression of IDO1 in various solid tumors is associated with poor prognosis (Yu et al., 2018). Interferon-γ or attachment-independent culture increased IDO1 expression in pancreatic cancer cells, leading to an increase in purine nucleotide production by facilitating the use of tryptophan as a source of formate. In addition to nucleotide synthesis in pancreatic cancer cells, formate has also been used in PSCs for nucleotide synthesis (Newman et al., 2021). These studies suggested that simultaneous alterations in metabolic status cooperatively promote pancreatic cancer progression (Figure 3).

## Impact of PSC on Therapy

Altered metabolism also contributes to resistance to therapeutic interventions. PSCs form dense fibrotic stroma, and deletion of PSCs by inhibitors of the hedgehog pathway improved gemcitabine delivery into pancreatic cancer tissue for a short time (Olive et al., 2009). The following study detected an increased amount of gemcitabine metabolites in PSCs, indicating their scavenging roles. Incubation of gemcitabine-containing culture medium with PSCs resulted in an increased amount of intracellular dFdCTP, an active metabolite of gemcitabine. This drug scavenging metabolic profile of PSCs lowers their availability to pancreatic cancer cells (Hessmann et al., 2018). Soluble factors from PSCs also affect gemcitabine metabolism. Conditioned medium from PSCs induced resistance to gemcitabine in pancreatic cancer cells. Fractionation of conditioned medium by high-performance liquid chromatography revealed that the protective small molecule was deoxycytidine. Deoxycytidine and gemcitabine compete for phosphorylation by deoxycytidine kinase, thereby attenuating the effect of gemcitabine (Dalin et al., 2019). However, the simple deletion of PSCs did not result in effective therapy for pancreatic cancer. Deletion of PSCs in a murine pancreatic cancer model led to the paradoxical promotion of cancer progression and shorter survival (Ozdemir et al., 2014). The following study identified a specific PSC population expressing the undifferentiated mesenchymal stem cell marker, meflin-positive PSCs (Mizutani et al., 2019). Meflin-positive PSCs were found to inhibit cancer progression. Therefore, such metabolic changes should be targeted by specific interventions aimed at cancer-promoting key points. Induction of quiescence of PSCs by vitamin D derivative, calcipotriol, is an adequate example (Sherman et al., 2014).

## Conclusion

This review discusses altered metabolic status in activated PSCs, effects on normal pancreatic cells, and cancer-promoting roles. Systemic metabolic/nutritional changes affect PSC function, and vice versa. In particular, PSC-induced metabolic reprogramming and coordinated metabolic coupling contribute to pancreatic cancer progression, which could be novel targets for therapeutic intervention. Further studies are warranted to clarify the complex regulation of metabolic interactions in pancreatic diseases.

## Author Contributions

SH, RM, and AM conceptualized the study design. SH and RM wrote the manuscript. AM supervised manuscript preparation. All authors have read and agreed to the published version of the manuscript.

## Funding

This work was supported by JSPS KAKENHI (19H03631 and 20K08300) and Smoking Research Foundation (to SH).

## Conflict of Interest

The authors declare that the research was conducted in the absence of any commercial or financial relationships that could be construed as a potential conflict of interest.

## Publisher’s Note

All claims expressed in this article are solely those of the authors and do not necessarily represent those of their affiliated organizations, or those of the publisher, the editors and the reviewers. Any product that may be evaluated in this article, or claim that may be made by its manufacturer, is not guaranteed or endorsed by the publisher.
